# Efficacy and effectiveness of case isolation and quarantine during a growing phase of the COVID-19 epidemic in Finland

**DOI:** 10.1038/s41598-022-27227-2

**Published:** 2023-01-06

**Authors:** Kari Auranen, Mikhail Shubin, Elina Erra, Sanna Isosomppi, Jukka Kontto, Tuija Leino, Timo Lukkarinen

**Affiliations:** 1 Department of Mathematics and Statistics, University of Turku, Vesilinnankatu 5, 20014 Turku, Finland; 2 Department of Clinical Medicine, University of Turku, Vesilinnankatu 5, 20014 Turku, Finland; 3 Department of Health Security, Finnish Institute for Health and Welfare, Mannerheimintie 166, 00271 Helsinki, Finland; 4 Epidemiological Operations Unit, City of Helsinki, Toinen linja 4 A, P.O. Box 6008, 00530 Helsinki, Finland

**Keywords:** Diseases, Medical research

## Abstract

Based on data collected as part of the contact tracing activity of the City of Helsinki Epidemiological Operations Unit, we evaluated the efficacy and effectiveness of isolating SARS-CoV-2 cases and quarantining their exposed contacts during a mildly growing phase of the COVID-19 epidemic in Finland in autumn 2020. Based on the observed symptom-to-symptom intervals in 1016 pairs of primary and secondary cases, we estimated that without case isolation or quarantine 40$\%$ (90$\%$ credible interval, CI 25–59) of transmission would have occurred on the day of or after symptom onset. One third of SARS-CoV-2 cases (*N* = 1521) had initially been quarantined, with a self-reported time until isolation (quarantine) of 0.8 days before symptom onset. This delay translates into an efficacy of 50$\%$ (90$\%$ CI 40–63) of averting secondary infections per quarantined case. Due to later isolation (mean 2.6 days after symptoms), the efficacy was smaller (24$\%$; 90$\%$ CI 12–41) in those two third of the cases (*N* = 3101) whose isolation was prompted by their symptoms, i.e. without being previously quarantined. At the population level, we evaluated the effectiveness of case isolation and quarantine on the growth rate of the COVID-19 epidemic in the autumn of 2020. Under a wide range of underlying assumptions, the rate would have been at least 2 times higher without case isolation and quarantine. The numbers needed to isolate or quarantine to prevent one secondary case were 2 and 20, respectively.

## Introduction

The second wave of the COVID-19 epidemic in Finland started in July 2020 after the incidence of SARS-CoV-2 infections had reached very low levels towards the end of the first wave^1^. In the next five months, the incidence of registered cases increased exponentially from approximately 1 to more than 50 per 100,000 per 2 weeks^2^. The first wave had been managed largely through social distancing as the number of daily social contacts was down to 30$\%$ of their normal level in the spring of 2020^3^. In the autumn the frequency of social contacts remained at approximately half of the normal level^4^. Schools operated relatively normally while there was a general recommendation to work from home and theatres and other public gatherings were subject to reduced capacity.

As of 14 February 2020, COVID-19 is defined as a generally hazardous disease by the Finnish Communicable Diseases Decree^5^. Each individual who tests positive for SARS-CoV-2 by either PCR or antigen laboratory test has to be notified to the National Infectious Disease Register and municipal public health units. During the study period, each case was contacted, set to isolation and interviewed for tracing the exposed individuals. Those meeting the criteria of high-risk exposure according to the national guidelines were contacted and quarantined. According to the national guidelines in place in 2020, testing was recommended only for those with symptoms compatible with SARS-CoV-2 infection. While the capacity of SARS-CoV-2 testing remained limited in the summer of 2020, it improved swiftly before the autumn and early winter.

It has been previously estimated that between 40$\%$ and 70$\%$ of SARS-CoV-2 transmission takes place before symptom onset^6,7^. This emphasises the importance of prompt identification and isolation of symptomatic cases as well as quick contact tracing to quarantine those exposed^8,9^. The effectiveness of case isolation and quarantine also depends on the proportion of exposed that are timely reached by contact tracing^8^. While efficient operation of municipal outbreak investigation is crucial, the success of contact tracing is ultimately affected by the willingness of the public to react at SARS-CoV-2 like symptoms and to adhere to isolation and quarantine rules^10^.

There are varying assessments of how much case isolation and quarantine have contributed to mitigating COVID-19 epidemics in different countries. In the UK, the effectiveness during autumn 2020 has been deemed small due to the poor adherence of the public to isolation and quarantine policies^10,11^. By contrast, it was estimated that case isolation and quarantine reduced the effective reproduction number as much as 35–45$\%$ in the early phase of the epidemic in New Zealand^9^. While reflecting true differences across areas, such estimates are prone to several underlying assumptions, including those about the proportion of transmission that occurs before symptoms and about the role of asymptomatic infections in maintaining virus circulation.

Here, we report empirical time lags in case isolation and quarantine during the second wave of the COVID-19 epidemic in Helsinki, the most populous community in Finland, from July 2020 through November 2020. Based on a large number of identified infector-infectee pairs, we then estimate the distribution of the time from symptoms to transmission (TOST) in the absence of case isolation and quarantine. Based on the time lags and the TOST distribution, we estimate the proportion of SARS-CoV-2 transmission that would take place after symptom onset without isolation and quarantine. We evaluate the efficacy of case isolation and quarantine as proportions of averted secondary infections, based on the upper tail of the TOST distribution and observed time lags until isolation/quarantine. Finally, we assess the effectiveness of case isolation and quarantine on the epidemic growth during the study period.

## Material and methods

### Study population

**Figure 1:**
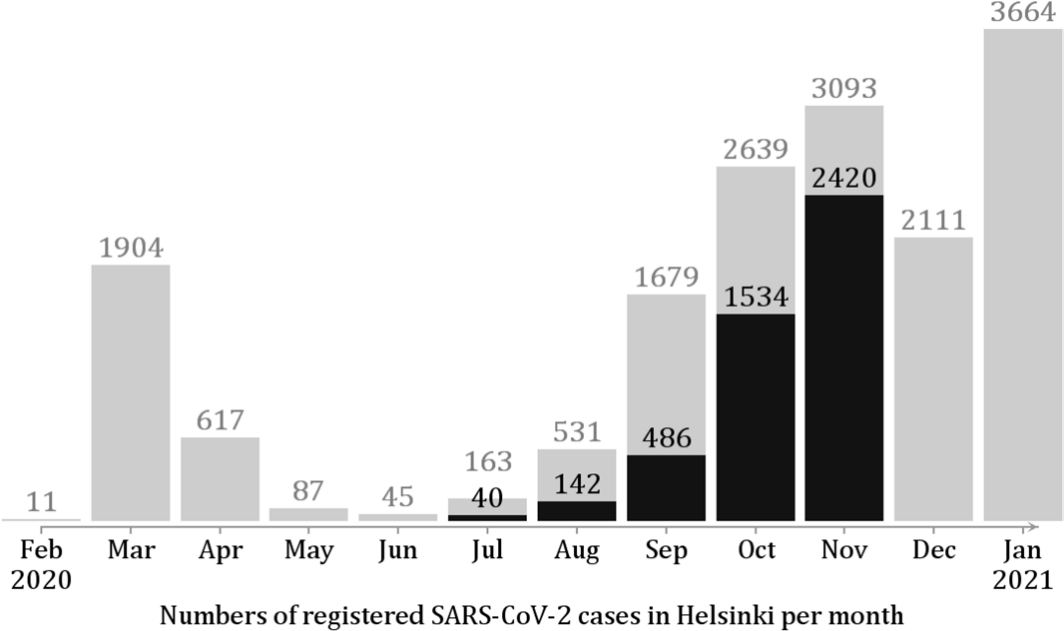
Monthly numbers of registered SARS-CoV-2 cases in the extended capital region of Helsinki and Uusimaa, February 2020–January 2021. The black bars shows the proportion of cases as analysed in this study, based on the SARS-CoV-2 cases in the City of Helsinki during the 5-month study period (Jul–Nov); see Table 1. The population of the HUS region is 1,650,000 and the population of Helsinki is 650,000. The average growth rate of the epidemic during the study period (Jul–Nov) was 3.0$\%$ per day, corresponding to an effective reproduction number 1.3.

The study population consists of all residents of the City of Helsinki with a population of approximately 650,000. The five-month study period from July 1 through November 30, 2020, covers the second wave of the COVID-19 epidemic in Finland until the exponential growth stopped due to newly intensified restriction measures (Fig. 1). During the study period, screening for asymptomatic infections was not common and screening-based SARS-CoV-2 cases were thus rare. The study period predated the availability of vaccines and the emergence in Finland of alpha (B.1.1.7) or other SARS-CoV-2 variants with increased transmission potential. The average growth rate of the epidemic during the study period was approximately 3% per day.

### Data sources and case identification

The data analysed in this study were retrieved from the SAI-COVID-19 database, a purposely built register based on the Communicable Diseases Act (1227/2016) for managing all SARS-CoV-2 contact tracing data on cases and their exposed contacts^12^. SARS-CoV-2 positive index cases were identified in a semi-automatised process in which all PCR-confirmed cases among Helsinki residents were reported to the City of Helsinki Epidemiological Operations Unit. The data were stored in a structural manner in the database as part of the normal work of the Unit. The variables used in this study are listed in Supplementary File A.

Cases identified through screening or with missing information on identity, date of symptom onset or self-isolation were discarded. Cases whose reported symptom onset was after registration or more than 25 days before registration were omitted as outliers.

### Time lags from symptoms to isolation

We describe the empirical distributions of four time lags relevant to isolation of the index cases: times from symptom onset to testing, registration, notification, and isolation call. In addition, we assess the distribution of time from symptoms to isolation in two complementary groups of index cases, those who were first identified as exposed and subsequently quarantined within $\pm$ 14 days from their own symptom onset (Group 1, quarantined), and those who were initially identified through their symptoms (Group 2, non-quarantined). In Group 2, the time of isolation is self-reported. In Group 1, the time of isolation is that of quarantining, or self-reported isolation if earlier.

### Exposed contacts and quarantine

We term as exposed all persons who had been in close contact (< 2 m for at least 15 min) with an index case during the index case’s estimated infectious period (1 day before symptom onset until end of September, and then 2 days). The length of the quarantine was 14 days until mid-October 2020, and 10 days thereafter.

### Timing of transmission

For each index case, if the source of infection was among the set of index cases and the case had earlier been reported as exposed, we formed a transmission pair consisting of a primary case (source) and a secondary case (exposed-turned-case). For each transmission pair, we defined the upper bound of the exposure window as the latest time transmission could have occurred, as reported by the secondary case.

We estimated the distribution of the time from symptom onset to transmission (TOST) in the absence of case isolation and quarantine, based on a likelihood function augmented with latent infection times of the primary case and taking into account right-truncation of transmission times due to case isolation/quarantine (Supplementary File B). We approximated the generation time distribution by the distribution of the serial interval, obtained as a convolution of the estimated TOST distribution and the distribution of the incubation period, taken from Lauer et al.^13^. The TOST distribution is needed to evaluate the averted proportion of secondary cases. The distribution of the generation interval is needed to evaluate the overall effectiveness of case isolation and quarantine (see Supplementary File C).

### Efficacy and effectiveness of case isolation and quarantine

We defined the efficacy of case isolation and quarantine in terms of averted proportions of secondary cases. Overall, we evaluated this proportion as $p_1\pi _Q + p_2 \pi _I$, where $p_1$ and $p_2$ (=$1-p_1$) are the proportions of index cases in Group 1 (quarantined) and Group 2 (non-quarantined), respectively, and $\pi _Q$ and $\pi _I$ are the proportions of averted infections by index cases in Groups 1 and 2, respectively. We assessed proportion $\pi _Q$ as

$$\begin{aligned} \pi _Q = \sum _{t=-20}^{20}Q(t)P_{tost}(t+0.5), \end{aligned}$$

where *Q*(*t*) is the proportion of exposed persons that isolated themselves (due to quarantine) on day *t* and $P_{tost}$ is the estimated tail probability of TOST. We calculated proportion $\pi _I$ similarly. For more details, see Supplementary File C.

We defined the effectiveness of case isolation and quarantine as their impact on the effective reproduction number (*R*), comparing the actual $R=1.3$ to the counterfactual $R_c$ in the absence of these activities. We derived the latter by modifying a previously presented relationship between the epidemic growth rate and the underlying reproduction number^8^. Given the relatively constant growth of the epidemic during the study period, we applied the relationship in a reverse manner, asking what the underlying (counterfactual) reproduction number would have been in the absence of case isolation and quarantine (see Supplementary File C for details). Apart from the distributions of the incubation and generation intervals, $R_c$ depends on coverage of case isolation (proportion of all symptomatic cases in the population that are tested and isolated), coverage of quarantine (proportion of all infectees in the population that are set to quarantine before symptom onset) and delays until isolation/quarantine. In addition, the proportion of those asymptomatically infected, with potentially reduced infectiousness, affects the level of $R_c$. Supplementary File C provides a detailed account of the assumed parameter values and their sources, as used also in a sensitivity analysis.

We evaluated the social cost of contact tracing in terms of the numbers of isolated and quarantined needed to prevent one case in the next generation of infection. These numbers were calculated as NNI=$1/(\pi _IR_c)$ and NNQ=$N_1/(N_2\pi _QR_c)$, respectively, where $N_1$ is the total number of quarantined and $N_2$ is the number of quarantined that were actually infected. These numbers address the immediate next-generation reduction in the number of secondary cases and thus omit the long-term (transmission-dynamic) implications.

### Ethics approval

This study analysed a historic dataset of SARS-CoV-2 epidemiologic registry. The data were collected as a normal contact tracing workflow in accordance with the Communicable Diseases Act (1227/2016) of Finland. All analyses were done in accordance with the EU General Data Protection Regulation and national legislation. The need to obtain direct informed consent was waived by the responsible data registrar as the data were not linked to any other information outside the registry nor were the subjects personally contacted in relation to this research. The data were analysed in a pseudonymised manner. No informed consent nor an ethical approval were needed.

## Results

### Cases

Altogether 5041 SARS-CoV-2 cases with residence in Helsinki were notified to the City of Helsinki Epidemiological Operations Unit between July 1 and November 30, 2020. After discarding cases identified through screening ($N=263$), or with missing identity code ($N=46$), with missing symptom onset time ($N=48$), with reported symptom onset time being after ($N=9$) or more than 25 days before ($N=10$) the registration time, with self-reported time of isolation being missing ($N=41$) or preceding the symptom onset by more than 7 days ($N=2$), altogether 4622 index cases (92$\%$) were retained in the analysis.

**Figure 2:**
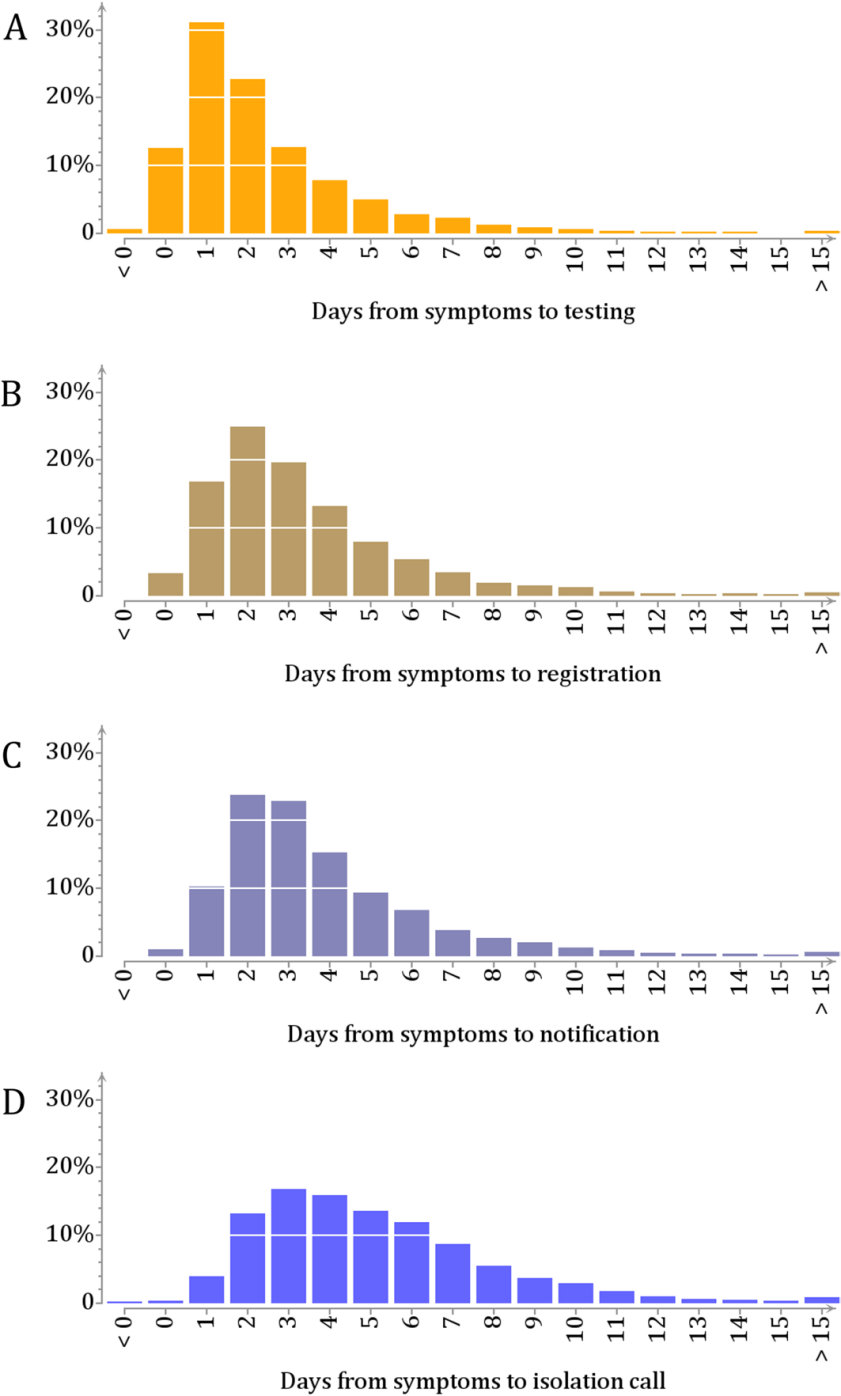
Time lags in case isolation. Based on 4622 SARS-CoV-2 cases, the figure shows the distributions of the time lag in days from symptoms to (**a**) testing (mean 2.4 days; median 2 days; (**b**) registration (mean 3.3 days; median 3 days); (**c**) notification (mean 3.8 days; median 3 days); and (**d**) isolation call (mean 5.0 days; median 5 day). The corresponding cumulative distributions of the time lags are shown in Supplementary Fig. 1.

The numbers of index cases increased by month, with 33$\%$ and 52$\%$ of the 4622 cases registered in October and November, reflecting the exponentially growing phase of the second epidemic wave (Fig. 1). Figure 2 shows the observed time lags from symptom onset to testing, registration, notification, and isolation call (the corresponding cumulative distributions are shown in Supplement A). There was a general trend towards shorter lags towards the end of the study period.

**Table 1:** Cases in Helsinki between July 1 and November 31, 2020.

	July	August	September	October	November	All 5 months
**A. Cases by source identity**						
Source among the cases (*N*)	0 (0$\%$)	21 (15$\%$)	124 (26$\%$)	443 (29$\%$)	663 (27$\%$)	1251 (27$\%$)
Source otherwise known/likely (*N*)	25	60	202	623	943	1853 (40$\%$)
Source not known (*N*)	15	61	160	468	814	1518 (33$\%$)
Total (*N*)	40	142	486	1534	2420	4622
**B. Cases by quarantine status**						
Group 1: Quarantined (*N*)	2	33	179	505	802	1521
Symptom to quarantine (mean, d)	5.5	1.2	1.2	-0.7	-1.4	-0.8
Group 2: Not quarantined (*N*)	38	109	307	1029	1618	3101
Symptom to isolation (mean, d)	4.8	5.4	3.7	2.6	2.2	2.6
Total (*N*)	40	142	486	1534	2420	4622

Panel A: numbers of cases by identity of infection source (source within the dataset/source otherwise known or likely/source unknown). Panel B: numbers of cases and the mean time until quarantine (Group 1) or isolation (Group 2). Group 1 includes cases who were quarantined within $\pm$ 14 days from symptom onset ($N=1521$). Group 2 includes the rest ($N=3101$).

For 27$\%$ (1251/4622) of the index cases, the source of infection was identified as another index case (Table 1A). By design, all source cases were residents of Helsinki. The source was known or likely known for an additional 40$\%$ (1853/4622) of the index cases and was unknown for 33$\%$ (1518/4622).

### Time lags from symptoms until isolation/quarantine

The 4614 index cases were divided into two groups according to whether or not they had been initially identified as exposed and, subsequently, quarantined within $\pm$ 14 days from symptom onset. Group 1 ($N=1521$; 33$\%$) thus consists of those quarantined before being registered as cases. Group 2 ($N=3101$; 67$\%$) involves cases that had no previous record of exposure.

**Figure 3:**
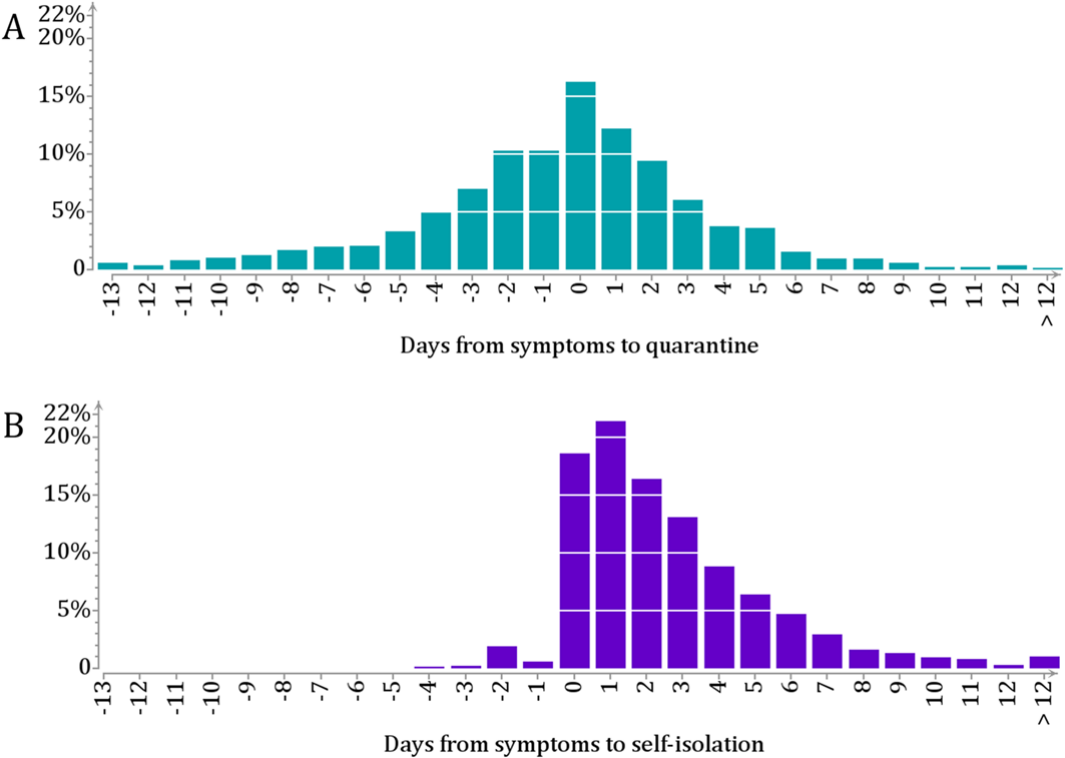
Time from symptoms to quarantine or self-isolation. (A) Time from symptom onset to quarantine onset in 1521 cases initially identified as exposed (Group 1). The mean time lag was $-0.8$ days. **(B)** Self-reported time from symptom onset to isolation in 3101 cases (Group 2). The mean time lag was 2.6 days.

Figure 3 shows the distribution of the self-reported time from symptom onset to quarantine (Group 1) or isolation (Group 2). In Group 1, the mean lag from symptoms to quarantine was $-0.8$ days (median 0, inter-quartile range IQR $[-2,~1]$). In Group 2, the mean lag from symptom to isolation was 2.6 days (median 2, IQR [1, 4]). The mean lags from symptom onset to quarantine or isolation decreased during the autumn and were always shorter in those quarantined (Table 1B). Of note, the mean lag from symptom onset to isolation in Group 2 was clearly shorter than the mean lag of 5 days from symptom to formal isolation call (Fig. 2D).

### Exposed contacts

The 4622 index cases had altogether 31837 contacts identified as exposed. The average number of contacts per case was thus 6.9 (median 3; range 0–183). Altogether 19% (859/4622) cases had no contacts identified and 17$\%$ (787/4622) had >10 contacts. Leaving out cases with >10 contacts, the average number of contacts per case was 2.4 (median 2).

**Table 2:** Exposed contacts.

	July	August	September	October	November	Total
N index case	40	143	488	1542	2430	4622
N exposed	307	1165	3288	8464	13,029	26,253
N quarantined	281	1087	3164	7538	11,999	24,069
N quarantined per index case	7.0	7.6	6.5	4.9	4.9	5.2
**N quarantined within ± 14 days**						
of symptom onset	6	37	216	476	694	1429
$\%$ of quarantined ($\%$)	2.1	3.4	6.8	6.3	5.8	5.9
Per index case	0.15	0.26	0.47	0.31	0.32	0.31

The table reports data for the 26,253 exposed contacts with Helsinki residence (the total number of exposed contacts was 31,837; see text). For each month, the table shows the number of index cases, exposed contacts, exposed individuals that were quarantined and their proportion per index case, and the number and proportion of quarantined that are identified as infected within ± 14 days of quarantine onset. In addition, the last line shows the number of exposed contacts that are identified as infected within ± 14 days of quarantine onset, per index case.

Contacts to Helsinki residents constituted 82$\%$ of all contacts made by the 4622 index cases. The average number of contacts per case to Helsinki residents was 5.7 (median 2; range 0–183). The number of exposed contacts per case was relatively stable over time (Table 2). Restricted to those with $\le 10$ contacts, the average was 2.5 contacts (median 2).

Of all 26,253 exposed contacts with residence in Helsinki, 92% (24,069) were set in quarantine. The proportion of quarantined recorded as cases within 14 days since quarantine onset was 5.9% (1429/24,069; Table 2).

### Time from symptom to transmission (TOST)

Altogether 1016 transmission pairs were identified by linking index cases (secondary cases) to previously recorded index cases (primary cases). The mean of the observed serial interval was 3.9 days (SD 3.1). The entire distribution of the observed serial interval is shown in Supplementary Fig. 2  (Supp. File B). The proportion of secondary cases quarantined or isolated within 4 days of quarantine or isolation of the index case was 69$\%$.

**Figure 4:**
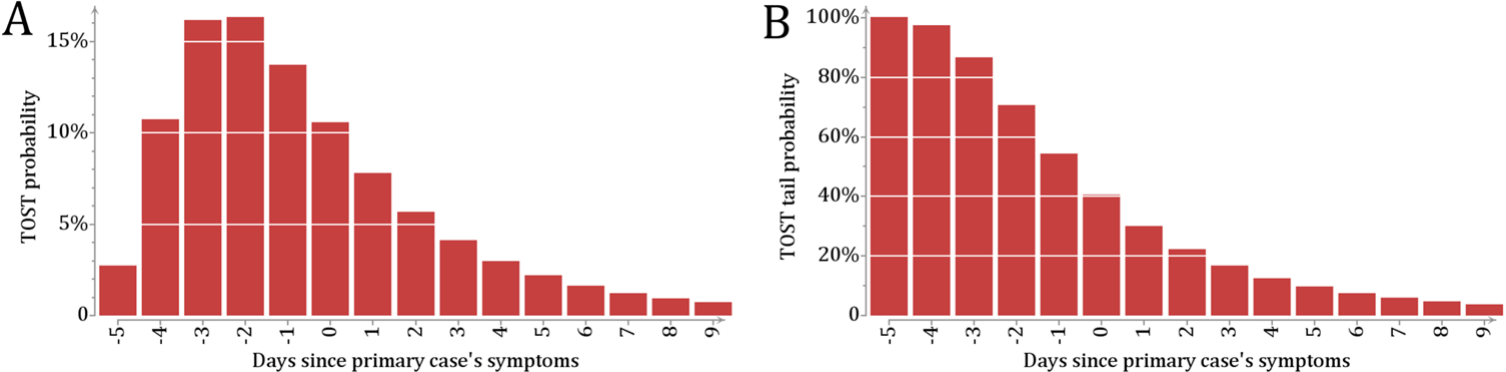
Timing of transmission. The figure shows the distribution of the time from symptom onset to transmission (TOST). **( A)** The estimated TOST distribution based on the observed serial intervals in 1016 transmission pairs. The figure presents the posterior predictive probabilities for transmission occurring on day $t=-5,\dots ,9$, since symptom onset. (** B**) The tail probabilities of the TOST distribution. This is interpreted as the probability that transmission occurs after day $t=-5,\ldots , 9$, since symptom onset.

Based on the observed serial intervals, we estimated the distribution of the time from symptom onset to transmission (TOST). Figure 4A shows the distribution by day since symptoms of the primary case. The median and mean of time from symptom onset to transmission (TOST) without case isolation and quarantine were -0.25 and 0.80 days, respectively. Figure 4B shows the corresponding tail probabilities, interpreted as the averted proportion of transmission, by day of isolation/quarantine. According to this analysis, isolation on the day of symptoms signifies a $40\%$ reduction in transmission (90$\%$ CI 25–59).

### Efficacy

Weighing the estimated TOST tail probabilities by the empirical distributions of the time lags from symptoms to quarantine (Group 1) and symptoms to isolation (Group 2), the averted proportion $\pi _Q$ of secondary cases was 50$\%$ (90$\%$ CI 40–63) per case if initially quarantined (Group 1), and $24\%$ (90$\%$ CI 12–41) per case if not quarantined (Group 2). The averted proportion $\pi _I$ of secondary cases per index case was thus $33\%$ (= 0.33*0.50 + 0.67*0.24; 90$\%$ CI 21–48).

**Figure 5:**
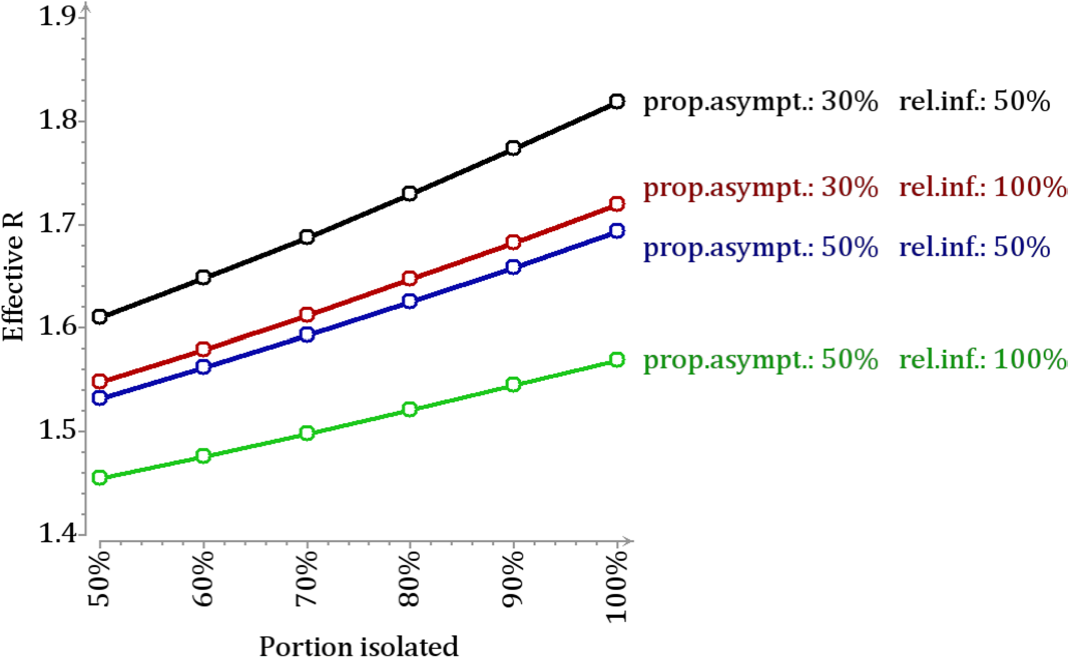
Effective R in the absence of case isolation and quarantine. The figure shows the counterfactual effective reproduction number $R_c$ under different assumptions about the coverage of case isolation (i.e. the proportion of all infected that were isolated; horizontal axis), the proportion of asymptomatic infections and the relative infectiousness of asymptomatically infected. The coverage of quarantine, i.e. the proportion of all exposed that were quarantined was assumed to be 33$\%$.

**Table 3:** Parameters and assumptions in the effectiveness model.

Parameter	Value/distribution	Source
Growth rate (*r*)	0.030 per day	Study population in autumn 2020 (see Suppl. File A)
Incubation time	Weibull (shape = 2.435, scale = 6.258)	^13^
Generation time	Suppl. Fig. 4 (Suppl. File B)	Approximated by the estimated serial interval
Coverage of isolation$^{\rm a}$ ($\epsilon _I$)	80$\%$/50$\%$	Good/poor adherence to testing (see Suppl. File C)
Mean delay until isolation ($\Delta _I$) since symptoms	2.6 days	This study (Table 1)
Coverage of quarantine$^{\rm b}$ ($\epsilon _T$)	33$\%$	Assumption
Mean delay until quarantine ($\Delta _T$) since infection	5 days	This study
Proportion of asymptomatic infections ($P_\alpha$)	30$\%$/50$\%$	30$\%$ = prop. of cases with unknown source (this study); 50$\%$^15^
Relative infectiousness of asymptotically infected ($x_{\alpha }$)	50$\%$/100$\%$	Assumption^7^

The table summarises the parameters used to evaluate the counterfactual effective reproduction number in the absence of case isolation and contact tracing. The parameter symbols refer to those as used in Supplementary File C.

$^\mathrm{a}$Proportion of symptomatic SARS-CoV-2 cases that are tested and isolated.

$^\mathrm{b}$Proportion of secondary cases set to quarantine before symptom onset due to contact tracing.

### Effectiveness

Assuming the proportion of asymptomatically infected was 30$\%$ (with 50$\%$ infectiousness) and the coverages of case isolation and quarantine were 80$\%$ and 33$\%$, respectively, the counterfactual effective reproduction number $R_c$ is 1.7. The corresponding epidemic growth rate is $r_c=0.087$ (per day). These values should be compared to the actual values of $R=1.3$ and $r=0.030$ in the study population in autumn 2020. As a sensitivity analysis, we calculated $R_c$ under a number of different assumptions (Table 3). With increasing coverage of isolation, $R_c$ increases (Fig. 5). Likewise, $R_c$ is the larger, the larger the assumed proportion of asymptomatically infected is, or the larger their relative infectiousness with respect to symptomatic cases is assumed to be. Nevertheless, under all scenarios the counterfactual reproduction number $R_c$ is between 1.4 and 1.8 (Fig. 5; Supplementary Table 1 in Supplementary File C).

With $R_c=1.7$, the number of isolated needed to prevent one case was 1.8 (NNI=$1/(\pi _IR_c$). The number of quarantined to prevent one case was 19 (NNQ=24069/ $(1521*\pi _QR_c)$). With $R_c=1.5$, these numbers are 2 and 21.

## Discussion

Case isolation, contact tracing and quarantining exposed persons have been widely used to mitigate the spread of SARS-CoV-2 during the early phases of the COVID-19 pandemic. Here, we assess the efficacy and effectiveness of isolation and contact tracing of SARS-CoV-2 cases during a mildly increasing phase of the COVID-19 epidemic in Helsinki, Finland, in autumn 2020. We report empirical time lags from symptom onset to isolation in SARS-CoV-2 cases. The lag was particularly short (mean −0.8 days) in those one third of the cases initially identified as exposed and subsequently quarantined. As we estimated that without isolation or quarantine about half of transmission would occur before symptom onset, the short time lag in quarantined cases amounts to cutting their infectiousness to half. The lag until self-isolation was longer (mean 2.6 days) in those two thirds of cases identified through symptoms, accounting for a reduction in infectiousness by one fourth. Overall, without isolation and quarantine the epidemic growth rate could have been two to three times higher, corresponding to an effective reproduction number 1.4–1.8 in the study population.

Based on 1016 transmission pairs of primary and secondary cases, the mean length of the observed serial (symptom-to-symptom) interval was 3.9 days. These empirical intervals were heavily truncated by the early isolation/quarantine of the primary cases. The data therefore disproportionately lacked transmission events occurring towards the end of the infectious period of the primary case. Adjusting for right truncation, we estimated that without case isolation and quarantine the median duration of the serial interval would have been 5.6 days, similar to the mean incubation time of 5.5 days^13^.

The similarity of the mean serial and incubation times suggests that transmission takes place at around symptom onset^14^. In agreement with this, we estimated that without isolation and quarantine 60$\%$ of SARS-CoV-2 transmission (90$\%$ CI 41–75) occurred before symptoms. Previously reported mean durations of the serial interval include 4.8 days^7^ and 5.1 (empirical intervals^6^), with estimated fractions of pre-symptom transmission of $67\%$ and $41\%$, respectively.

The TOST distribution (Fig. 4) describes the variability in the timing of transmission around the symptom onset as it would occur without isolation or quarantine. Accordingly, we assessed the proportions of averted infections by weighing the daily TOST tail probabilities by the observed daily proportions of quarantine or isolation onset. Among the one third of cases first identified as exposed and subsequently quarantined, this amounted to a 50$\%$ reduction in the number of secondary cases (90$\%$ CI 40–63). The remaining two thirds of cases isolated themselves later with respect to the symptom onset, corresponding to an estimated 24$\%$ reduction in the number of secondary cases (90$\%$ CI 12–41).

We evaluated the effectiveness of isolation and quarantine in terms of their impact on the epidemic growth. In particular, without isolation or quarantine the COVID-19 epidemic would have grown considerably faster, and instead of 3 weeks, the epidemic doubling time in the study population would have been 1–2 weeks, corresponding to an effective reproduction number of 1.5–1.7. These inferences agree with the estimated basic reproduction number of the original SARS-CoV-2 strain being approximately 2.5 to 3.5^15,16^ and the frequency of social contacts in the study population remaining at approximately half of their normal level in autumn 2020^4^.

The secondary attack rate (SAR), defined as the proportion of exposed found SARS-CoV-2 positive within 14 days since quarantine onset, was 6.1$\%$. In the UK, the corresponding figure in autumn 2020 was 6.9$\%$ (close contacts)^17^. Because of the quarantine onset broadly coincided with the symptom onset, the SAR in our material was very similar to the proportion (5.9$\%$) of those quarantined within 14 days of symptom onset.

Based on a model of SARS-CoV-2 transmission, James et al. showed an almost linear relationship between the reduction in the (effective) reproduction number and the proportion of secondary cases that were isolated/quarantined within 4 days from the isolation of the primary case^9^. With our estimate of a 69$\%$ proportion, the reduction in the (effective) reproduction number should be around 30–40$\%$.

The social cost of isolation can be evaluated in terms of the numbers needed to isolate (NNI) or quarantine (NNQ) to prevent one secondary case in the generation of infection. We evaluated that NNI and NNQ were approximately 2 and 20. For example, with 24,000 quarantined individuals, NNQ of 20 corresponds to approximately 1200 averted infections in the next generations of infection during autumn 2020. In practice, the costs are much more favourable as the above-mentioned figures do not acknowledge the fact that each averted case means an entire chain of onward transmission being prevented (cf.^18^). While such an analysis would be needed for a proper cost-effectiveness analysis, it is plausible that an epidemic surge would have induced mitigation measures instigated earlier than in late November 2020.

There are a number of caveats in our study. First, while the early isolation and quarantine as reported in this study are hallmarks of efficacious action, at the same time they created an analytical problem as long serial intervals tend to be truncated from the sample. Because of this, we could not use a heavy-tailed symmetric TOST distribution earlier found as best describing the duration of incubation^6^. We used the lognormal distribution, which may favour inferring transmission long after symptom onset. To address this concern and the discrete (daily) scale of the observed symptom onset times, we read the TOST distribution with a shift of 0.5 days. Our analysis also relies heavily on the assumed distribution of the incubation time. Assuming a shorter incubation period than the one used in the current study would have led to more favourable efficacy and vice versa.

Second, our inferences about the averted proportion of transmission largely depend on relying on self-reported information about early isolation. In particular, the time lags from symptom onset to self-isolation were shorter than the lags from symptom onset to the administrative isolation call. If isolation would have commenced only at the call or the adherence to isolation and quarantine would have been poor, our assessment of the efficacy and effectiveness would be more pessimistic.

Third, our assessment of the population-level effectiveness of case isolation and quarantine depends on a number of assumptions not directly derivable from the material. For example, the proportion of asymptomatic infections or the proportion of fully infectious individuals identified by contact tracing affect the estimate of the counterfactual epidemic growth rate. Nevertheless, under a wide range of assumptions, the rate would have been much larger without isolation and quarantine.

The dataset was derived from a time period before the emergence of the alpha variant (B.1.1.7) or later variants of SARS-CoV-2 in Finland. Due to e.g. the increased transmissibility of later SARS-CoV-2 variants, the results cannot be directly applied to later stages of the pandemic.

In summary, our analysis of a large comprehensive dataset corroborates earlier findings on that a considerable portion of SARS-CoV-2 transmission takes place before symptom onset. Prompt isolation of symptomatic cases and their exposed contacts is essential to reduce the pace of the epidemic. If most citizens self-isolate early by symptom onset or exposure to the virus, the epidemic growth can be considerably moderated. This, however, requires that the effective reproduction number remains close to one, which may not be eventually possible when new variants with larger reproduction numbers and shorter incubation periods have emerged.

## Supplementary Information

Supplementary Information.

## Data Availability

The data are confidential according to the Finnish legislation. The analysis code to estimate the serial interval and to assess the effectiveness of case isolation and contact tracing is available from the principal author upon request.
