# Supplementary material for: Efficacy and effectiveness of case isolation and quarantine during a growing phase of the COVID-19 epidemic in Finland
Source: Sci Rep. 2023 Jan 6;13:298. doi: 10.1038/s41598-022-27227-2 (PMC9817446; doi:10.1038/s41598-022-27227-2)
Supplement: Supplementary file 1 — Supplementary Information. [file 41598_2022_27227_MOESM1_ESM.pdf]

Supplementary File A

The following data items were retrieved for all 5041 index cases and their 31837 exposed contacts from the SAI-COVID-19 database. This database is a purposely built register to manage data on all SARS-COV-2 data on the identified SARS-CoV-2 positive cases and their exposed contacts.

Index cases

Variable	Explanation/remarks
Personal identification code	Unique pseudonymised identity of the index case
Community of residence	Used to select Helsinki residents
Symptoms at testing (yes/no)	Used to exclude screening-based cases
Date of symptom onset	Self-reported time (day) of symptom onset
Date of virus testing	
Date of registration	Day when the case was registered
Date of notification	Day when the Epidemiological Operations Unit was notified of the case
Date of isolation call	Day when the case was first contacted by the Epidemiological Operations Unit
Date of isolation	Self-reported time (day) when the case isolated himself/herself
Source of infection	Identity of the infector; used to link transmission pairs
Certainty of exposure	Whether the source was known (i.e. identified with high certainty), or likely known

Exposed contacts

Variable	Explanation/remarks
Personal identification code	Unique pseudonymised identity of the exposed individual
Community of residence (postal code)	Used to select Helsinki residents
Date of last exposure	Used to define the upper bound of the exposure window
Source of exposure	The identity of whom the individual had been exposed to
Date of quarantine onset	The self-reported time (day) when the exposed individual isolated himself/herself

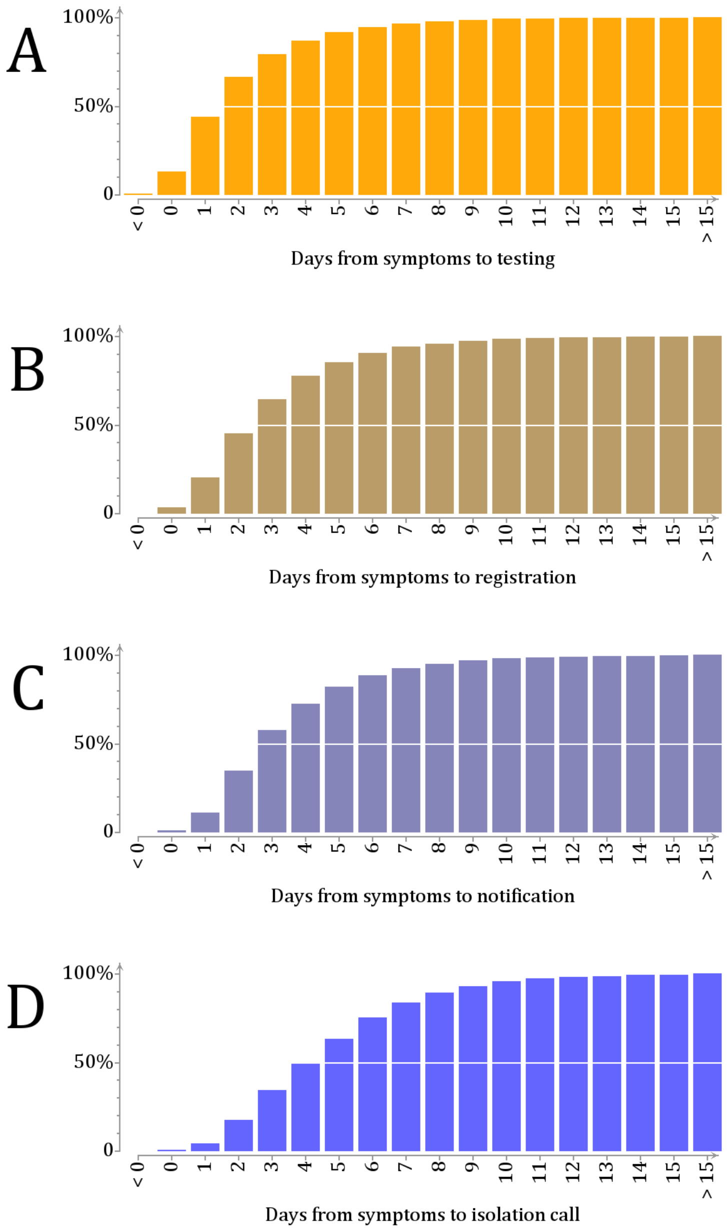

Supplementary Figure 1. Time lags in case isolation. Based on 4622 SARS-CoV-2 cases, the figure shows the distributions of the time lag in days from symptoms to (a) testing; (b) registration; (c) notification; and (d) isolation call. The data are the same as in Figure 2 of the main text but here presented as cumulative distributions.

Supplementary File B

A total of $N = 1016$ transmission pairs were formed by linking index cases through the indicated sources of infection. Let $t_{j1}^{(s)}$ and $t_{j2}^{(s)}$ denote the observed symptom onset times of the primary and secondary cases in transmission pair j. Supplementary Figure 2 shows the distribution of the observed serial interval, i.e. of the differences $t_{j2}^{(s)} - t_{j1}^{(s)}$, $i = 1, ..., N$.

Let $t_{j2}^{(i)}$, $i = 1, \ldots, N$, denote the infection times of the secondary cases. We treat these unobserved times as model unknowns, in addition to the parameters θ of the distribution of TOST (time from symptom onset $t_{j1}^{(s)}$ to transmission at $t_{j2}^{(i)}$). The complete-data likelihood contribution from transmission pair j is then

$$
\begin{aligned}
L(\theta, t_{j2}^{(i)}) &= p(t_{j2}^{(s)}, t_{j2}^{(i)} | \theta, t_{j1}^{(s)}) = p(t_{j2}^{(i)} | \theta, t_{j1}^{(s)}) \times p(t_{j2}^{(s)} | t_{j2}^{(i)}) \\
&= \frac{p_{tost}(t_{j2}^{(i)} - t_{j1}^{(s)} | \theta)}{\int_{-\infty}^{u_j} p_{tost}(u - t_{j1}^{(s)} | \theta) du} \times p_{incub}(t_{j2}^{(s)} - t_{j2}^{(i)}).
\end{aligned}
$$

The above expression takes into account right truncation, i.e. the fact that only transmissions occurring before the isolation of the primary case are included in the analysis data set.

Right truncation means that long TOST durations tend to be underrepresented in the sample of transmission pairs, because onward transmission occurring during the later part of the primary case's period of infectiousness remain more likely unrealised due to isolation or quarantine of the primary case. Therefore, the likelihood contribution of each transmission pair needs to be conditioned on the event that infection occurred before the isolation or quarantine of the primary case. This time, denoted by u_j for pair j, was obtained as the isolation or quarantine time of the primary case, which ever occured earlier.

The density $p_{incub}(\cdot)$ of the incubation time is assumed to be Weibull with shape 2.453 and scale 6.258 [1]. The model unknowns then include the parameters θ of the TOST distribution (see below) and the unknown infection times. Their joint Bayesian posterior density is proportional to

$$
\prod_{j=1}^{N} L(\theta, t_{j2}^{(i)}) \times p(\theta),
$$

where $p(\theta)$is the prior distribution of the parameters. The posterior of θ is conditioned on the symptom onset times $(t_{j1}^{(s)}, t_{j2}^{(s)})$, $1, ..., N$. For simplicity of analysis, we have here omitted potential dependencies between transmission pairs, e.g. situations where two secondary cases may have shared the same primary case.

We parameterised the TOST distribution as a shifted lognormal distribution with parameters d (shift), σ (scale) and m (median). Variable $\tau_j^{(tost)} = t_{j2}^{(i)} - t_{j1}^{(s)}$ then has density

$$\begin{aligned} p_{tost}(\tau_j^{(tost)}|d,\sigma,m) &= \text{LogNormal}(t_j^{(tost)} - d|d,\sigma,m) \\ &= \tfrac{1}{(\tau_j^{(tost)}-d)\sqrt{2\pi}\sigma} \exp\left(-(\log(\tau_j^{(tost)} - d) - \log(m-d))^2/(2\sigma^2)\right). \end{aligned}$$

We took the prior density of the three unknown parameters to be

$$\begin{aligned} p(\theta) &= p(d)p(\sigma)p(m) \\ &= \text{Exp}(-(d+5)|\text{rate}=1) \times \text{Gamma}(\sigma|\text{shape}=4, \text{rate}=2)\times \\ &\quad \text{Normal}(\mu|\text{mean}=0, \text{var}=16),\ d \le -5,\ \sigma > 0. \end{aligned}$$

Finally, we explored the posterior of the model unknowns, conditioning on the observed symptom times $(t_{j1}^{(s)}, t_{j2}^{(s)})$, $j = 1, ..., N$. Supplementary Figure 3 shows the marginal posterior distributions of parameters d, σ and μ, realised using a Markov chain Monte Carlo sampling. The posterior means of the three parameters were $\hat{d} = -5.42$, $\hat{\sigma} = 0.53$, and $\hat{m} = -0.07$.

Supplementary Figure 4 shows the posterior predictive distributions of the mean of the empirical serial interval, i.e. the serial interval that is subject to the pattern of truncation as in the actual data. In addition, the corresponding predictive distribution of tail probability at 7 days is shown.

Supplementary Figure 5 shows the density of the estimated serial interval in the absence of case isolation and quarantine. We obtained the distribution as a convolution of the density of the incubation period and the density of TOST duration as estimated in this study:

$$\begin{aligned} p_{serial}(t) &= \textstyle\int_0^\infty p_{incub}(u)p_{tost}(t-u)du \\ &= \textstyle\int_0^\infty [\text{Weibull}(u|\text{shape}=2.453, \text{scale}=6.258) \\ &\qquad \times\text{LogNormal}(t-u-d|d=-5.42, \sigma=0.53, d=-0.07)]\, du. \end{aligned}$$

The distribution is used as a proxy of the distribution of generation time interval in assessing the effectiveness (see Supplementary File C).

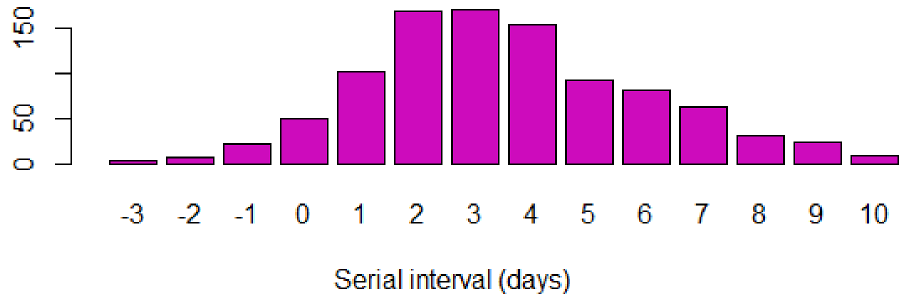

Supplementary Figure 2. Observed serial interval. The figure shows the observed distribution of the symptom-to-symptom times in 1016 transmission pairs. The mean of this empirical distribution is 3.9 days (SD 3.1). The proportion of negative serial intervals was 3.1%.

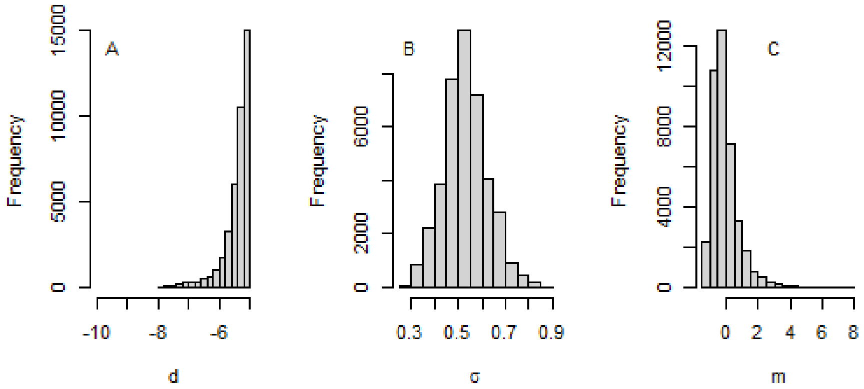

Supplementary Figure 3. Posterior distributions of parameters d (shift, σ (scale) and m (median) of the shifted lognormal TOST distribution. The marginal posteriors of the three parameters are based on 40,000 Markov chain Monte Carlo samples from the joint posterior distribution of the model unknowns, involving the latent transmission times and realised by a Metropolis-Hastings algorithm.

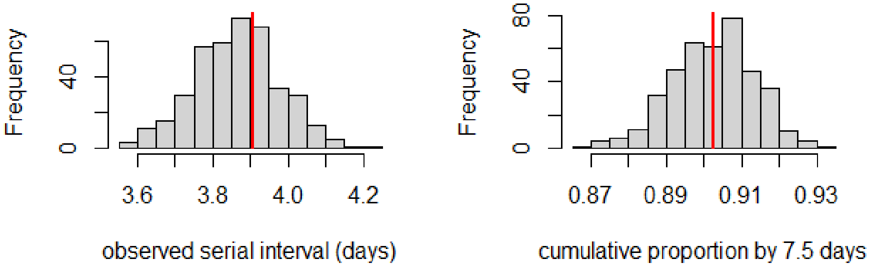

Supplementary Figure 4. Posterior predictive distributions of (A) the mean of the empirical (observed) serial interval, i.e. the serial interval that is subject to the pattern of truncation as in the actual data; (B) the cumulative proportion of individuals with duration of the observed serial $\leq$ 7.5 days. The actually observed values are shown by the red lines. The posterior distributions are based on a sample of size 40,000 from the posterior of the parameters of the TOST distribution.

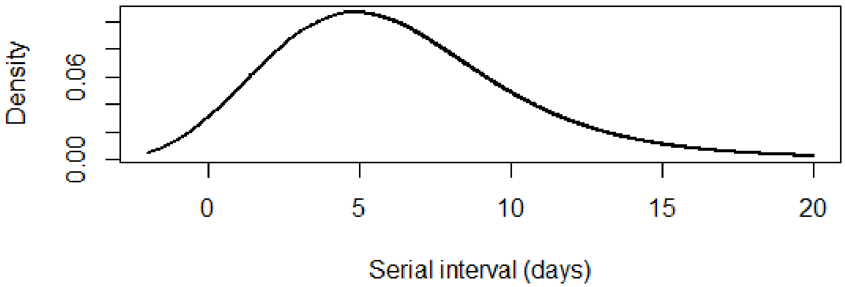

Supplementary Figure 5. Serial interval. The figure shows the density of the serial interval. This distribution pertains to a counterfactual situation in the absence of case isolation or contact tracing. The median and mean of this serial interval are 5.6 and 6.2 days, respectively.

References

[1] Lauer SA, Grantz KH, Bi Q, Jones FK, Zheng Q, Meredith HR, et al. The incubation period of coronavirus disease 2019 (COVID-19) from publicly reported confirmed cases: estimation and application. Ann Intern Med. 2020; https://doi.org/10.7326/M20-0504.

Supplementary File C

Efficacy. We defined the efficacy of case isolation as the expected proportion of secondary cases averted by isolation of a symptomatic SARS-CoV-2 case. We calculated this proportion as

$$\pi_I = \sum_{t=-20}^{20} I(t) P_{tost}(t+\delta),$$

where $I(t)$ is the proportion of cases that isolated on day t since symptom onset (Figure 3B in the main text) and $P_{tost}(t)$ is the tail probability of the TOST (time from symptoms to transmission) distribution on day t (Figure 4B in the main text). We made a shift of $\delta = 0.5$ days to account for the discrete nature of the observed times to isolation.

We defined the efficacy of quarantine as the expected proportion of secondary cases averted due to quarantining an infected individual initially identified as an exposed contact (i.e. before symptom onset). We calculated this proportion as

$$\pi_Q = \sum_{t=-20}^{20} Q(t) P_{tost}(t+\delta),$$

where $Q(t)$ is the proportion of the exposed that isolate due to quarantine on day t since symptom onset (Figure 3A). Also here we used a shift of $\delta = 0.5$ days. We calculated the combined efficacy of case isolation and quarantine as a weighted average

$$p_1 \pi_Q + (1 - p_1) \pi_I,$$

where $p_1 = 0.33$ is the proportion of cases that were first identified as exposed (Table 1B).

Effectiveness. We evaluated the effectiveness of case isolation and quarantine by calculating what the effective reproduction number R would have been in the absence of these policies. To this end, we applied a relationship between R in the absence of case isolation and quarantine, and the growth rate r when these activities are in place. Specifically, Ferretti et al. showed that, given R, the epidemic growth rate r under case isolation and quarantine can be found as the solution of the following eigenvalue problem [1]:

$$y(\tau) = Rg(\tau)e^{-r\tau}[1-\epsilon_I s(\tau-\Delta_I)] \int_0^\infty \left(1 - \epsilon_T + \epsilon_T \frac{1 - s(\rho + \tau - \Delta_T)}{1 - s(\rho - \Delta_T)}\right) y(\rho) d\rho. \quad (E1)$$

Here $g(\tau)$ is the density of the generation interval at duration τ since infection and $s(\tau)$ is basically the probability showing symptoms by time τ since infection, i.e. the cumulative distribution of the incubation time (see below). Equation (E1) is a modification of that of Ferretti et al. (see their supplementary equations (24) and (25)), here including the contributions of time lags

from symptom onset to isolation (Δ_I) and quarantine (Δ_T). Another difference to how Ferretti et al. applied relation (E1) is that we determined the underlying reproduction number R given the current (i.e. observed) growth rate r (see below). Of note, in our context such a counterfactual reproduction number pertains to a population with social distancing in place but very low level of pre-existing immunity [2]. We approximated $g(\tau)$ by the distribution of the serial interval (Supplementary Figure 5 in Supplementary File B). The expectations of the two distributions are the same although the approximation will likely overestimate the variability in generation times [3].

Parameter ϵ_I is the coverage of case isolation, i.e. the proportion of symptomatic SARS-CoV-2 cases that are tested and subsequently isolate themselves with a mean time lag $\Delta_I = 2.6$ days since symptom onset (Figure 3B). Parameter ϵ_T is the coverage of quarantine, i.e. the proportion of secondary SARS-CoV-2 cases that are quarantined with a mean time lag Δ_T since their infection. We set $\Delta_T = 5$ days, which is approximately the sum of the mean incubation time (5.5 days) and the mean time from symptoms until quarantine onset (-0.8 days; Figure 3A).

We set ϵ_I to either 0.80 or 0.50, corresponding to good or poor adherence to the testing policy, respectively. We set ϵ_T to 0.33, which is the proportion of all registered cases that were quarantined in our material. Finally,

$$s(\tau) = \frac{1 - P_\alpha}{1 - P_\alpha + P_\alpha x_\alpha} \int_0^\tau P_{incub}(u)du,$$

where P_α is the proportion of asymptomatically infected with relative infectiousness x_α and $P_{incub}(u)$ is the probability the symptoms show by time u since infection (i.e. the cumulative distribution of the incubation period); see Ferretti et al. [1]. We set P_α to either 0.50 or 0.30. Combined with $\epsilon_I = 0.80$, these choices correspond to assuming that 56% or 40%, respectively, of all infections are assumed to be registered (Supplementary Figure 6). We assume that contact tracing leads to quarantining proportion $\epsilon_T = 0.33$ of those with asymptomatic infection.

The epidemic growth rate r during the study period was estimated from the daily numbers of SARS-COV-2 cases in the study area (Helsinki, [4]) and in the larger, extended capital region. A simple liner regression model was fitted to log-transformed daily numbers with time as the explanatory variable. The estimated growth rate was approximately 3% per day, with some variability in the estimate depending on whether the first two months with few cases were included or not. The estimate from the larger area was 2.8% per day, with even more consistent exponentially growing pattern of the epidemic. In our analysis, we used the rough value of 3.0% per day.

While Ferretti et al. applied relation (E1) to calculate epidemic growth rates given the current reproduction number, the authors pointed out that the relationship can be used the other way round: given a specific value of r, one can find what value R would have had without the case isolation and quarantine policies [1]. We applied the relationship in this manner to find

the counterfactual R_c there would have been in the absence of case isolation and quarantine. This was based on the notion that the steady exponential growth of the epidemic during the study period means that also the corresponding effective reproduction number was constant. The same then applies to the counterfactual reproduction number. Both the actual and counterfactual reproduction numbers can be viewed as effective reproduction numbers as both pertain to a population in which social distancing was widely exercised. For each combintation of the parameters, once R_c was found, the corresponding growth rate was determined by applying relation (E1) in the absence of case isolation and quarantine by setting the coverages of case isolation and quarantine to zero.

Table 3 of the main text summarises the parameters and their sources. Supplementary Table 1 shows the values of the effective reproduction number and the corresponding epidemic growth rates in the absence of case isolation and quarantine under some specific choices about the coverage of case isolation and quarantine and about the asymptomatic infections. The scenarios are special cases of those presented in Figure 5 of the main text.

References

[1] Ferretti L, Wymant C, Kendall M, Zhao L, Nurtay A, Abeler-Dörner, et al. Quantifying SARS-CoV-2 transmission suggests epidemic control with digital contact tracing. Science. 2020; 368: 619.

[2] Ahava MJ, Jarva H, Jääskeläinen AJ, Lappalainen M, Vapalahti O, Kurkela S. Rapid increase in SARS-CoV-2 seroprevalence during the emergence of Omicron variant, Finland. Eur J Clin Microbiol. 2022; 41;997-999.

[3] Lehtinen S, Ashcroft P, Bonhoeffer S. On the relationship of serial interval, infectiousness profile and generation time. J R Soc Interface. 2021; 18:20200756. https://doi.org/10.1098/rsif.2020.0756.

[4] Registered daily numbers of SARS-CoV-2 cases in Helsinki, Finland (in Finnish). 2022. In: https://www.hel.fi/helsinki/korona-fi/sote-palvelut/korona-tilanne/tietoja-koronatilanteesta. Accessed 19 November, 2022.

Supplementary Table 1. The table summarises sets of alternative parameter assumptions when evaluating the (counterfactual) effective reproduction number R_c and the corresponding epidemic growth rate r in the absence of case isolation and quarantine in the study population in autumn 2020. The actual values were $R = 1.3$ and $r = 0.030$ per day.

% of asymptomatic infections P_α	Relative infectiousness of asymp. infected x_α	Coverage of case isolation ϵ_I	Coverage of quarantine ϵ_T	R_c	Growth rate r (/day)
0.30	0.50	0.80	0.33	1.73	0.087
0.30	1.00	0.80	0.33	1.65	0.078
0.30	0.50	0.50	0.33	1.61	0.073
0.30	1.00	0.50	0.33	1.55	0.066
0.50	0.50	0.80	0.33	1.62	0.075
0.50	1.00	0.80	0.33	1.52	0.063
0.50	0.50	0.50	0.33	1.53	0.064
0.50	1.00	0.50	0.33	1.45	0.055

A	B	C
Asymptomatically infected, with infectiousness 50% or 100%	Cases not isolated/ isolating, yet fully infectious cases	Cases identified by contact tracing and isolated/isolating (Groups 1 and 2 in the data)
30%	14% = 0.20*0.70	56% = 0.80*0.70

Supplementary Figure 6 Proportions of (A) asymptomatically infected cases (P_α); (B) symptomatically infected cases who are not tested or identified through contact tracing ($(1-\epsilon_I)(1-P_\alpha)$); these cases are assumed *not to* isolate; (C) symptomatically infected, tested and isolated cases ($\epsilon_I(1-P_\alpha)$). Under the base-case model, the proportions are as shown in the figure. Assuming $P_\alpha = 0.50$ and $\epsilon_I = 0.80$, the proportions are 0.50,0.10 and 0.40. Assuming $P_\alpha = 0.30$ and $\epsilon_I = 0.50$, the proportions are 0.30,0.35 and 0.35
